# Draft Genome Sequence of *Lactobacillus curvatus* FLEC03, a Meat-Borne Isolate from Beef Carpaccio Packaged in a Modified Atmosphere

**DOI:** 10.1128/genomeA.00584-17

**Published:** 2017-06-29

**Authors:** Lucrecia C. Terán, Gwendoline Coeuret, Raúl Raya, Marie-Christine Champomier-Vergès, Stéphane Chaillou

**Affiliations:** a MICALIS Institute, INRA, AgroParisTech, Université Paris-Saclay, Domaine de Vilvert, Jouy-en-Josas, France; b CERELA-CONICET, Centro de Referencia para Lactobacilos, San Miguel de Tucumán, Argentina

## Abstract

In this study, we present the draft genome sequence for *Lactobacillus curvatus* FLEC03. This strain was isolated from beef carpaccio packaged in a modified atmosphere. The draft genome will contribute to understanding the role of *L. curvatus* strains in food products (fermentation, biopreservation, or spoilage) through comparative genomics with other strains.

## GENOME ANNOUNCEMENT

*Lactobacillus curvatus* is a lactic acid bacterium that was first described in the 1970s and was first associated with fermented meat products (1, 2). The species *L. curvatus* is phylogenetically related to *L. sakei*, *L. fuchuensis*, and *L. graminis* (3), which belong to the *L. sakei* clade of psychrotrophic* Lactobacillus* spp. To date, three complete genome sequences and seven draft genome sequences of *L. curvatus* strains are available in GenBank. All of these strains originate from a wide variety of food products, including fermented sausage (4), sushi (5), milk (6), vegetable marinade (7), and kimchi (8).

*L. curvatus* FLEC03 was isolated in 2008 from a slice of beef carpaccio after 7 days of storage at 8°C, a type of food product and storage condition where this species is often identified (9). This strain was found dominant in the meat slice, having reached a population level of 10^7^ CFU g^−1^, although the meat did not show any characteristics of spoilage. The genome sequencing of this strain was undertaken for the purpose of genomic comparison with other strains, in order to get better insight into the genomic repertoire of the *L. curvatus* species in relation to meat fermentation, biopreservation, or spoilage.

The whole-genome sequencing of *L. curvatus* FLEC03 was carried out by Eurofins MWG Operon Laboratories (Ebersberg, Germany) using Illumina MiSeq 2 × 150-bp paired-end libraries. The 1.45 million reads were assembled *de novo* using Velvet software (10) after choosing the best *k*-mer value of 85. The draft assembly resulted in 46 contigs from 1,717 to 307,439 bp (*N*_50_ of 85,282 bp). The contigs were aligned against the *L. sakei* 23-K complete genome using progressiveMauve (11) to give two high-quality scaffolds. The first scaffold (1,854,704 bp, coverage of 62×), comprising 40 contigs with an overall G+C content of 41.82%, was for the chromosome sequence. The second scaffold (46,973 bp, coverage of 116×), comprising 6 contigs with a G+C content of 37.87%, was for the plasmid pFLEC03A1 sequence. Annotation performed using the MicroScope platform (12) detected 1,926 coding sequences (CDSs), 40 pseudogenes, 3 rRNAs, and 30 tRNAs for the chromosome and 59 CDSs for the plasmid. Manual curation of the annotated sequences confirmed the presence in the chromosome of one prophage region of 32,532 bp and one CRISPR-Cas system of type II. Bacteriocin cluster genes were not detected using Bagel version 3.0 (13), indicating that the preservative potential of this strain is not correlated to bacteriocin production. Unlike other *L. curvatus* strains sequenced so far, strain FLEC03 is also characterized by the presence of 8 cell-surface multicomponent complexes (14) similar to those present in *L. sakei* strain 23K (15) and whose functions are related to bacterial adhesion and complex carbohydrate molecule scavenging. The FLEC03 strain has been deposited in the CIP Culture Collection under the reference CIP 110935. 

### Accession number(s).

This whole-genome shotgun project has been deposited in ENA under BioProject number PRJEB20186 and the assembly under the accession numbers FXDK01000001 to FXDK01000046. The versions described in this paper are the first versions.

## References

[1] Kandler O 1970 Amino acid sequence of the murein and taxonomy of the genera *Lactobacillus*, *Bifidobacterium*, *Leuconostoc* and *Pediococcus*. Int J Syst Evol Microbiol 20:491–507. doi:10.1099/00207713-20-4-491.

[2] Hammes WP, Bantleon A, Min S 1990 Lactic acid bacteria in meat fermentation. FEMS Microbiol Lett 87:165–174. doi:10.1111/j.1574-6968.1990.tb04886.x.

[3] Zheng J, Ruan L, Sun M, Gänzle M 2015 A genomic view of lactobacilli and pediococci demonstrates that phylogeny matches ecology and physiology. Appl Environ Microbiol 81:7233–7243. doi:10.1128/AEM.02116-15. 26253671 PMC4579461

[4] Hebert EM, Saavedra L, Taranto MP, Mozzi F, Magni C, Nader MEF, Font de Valdez G, Sesma F, Vignolo G, Raya RR 2012 Genome sequence of the bacteriocin-producing *Lactobacillus curvatus* strain CRL705. J Bacteriol 194:538–539. doi:10.1128/JB.06416-11. 22207745 PMC3256670

[5] Cousin FJ, Lynch SM, Harris HMB, McCann A, Lynch DB, Neville BA, Irisawa T, Okada S, Endo A, O’Toole PW 2015 Detection and genomic characterization of motility in *Lactobacillus curvatus*: confirmation of motility in a species outside the *Lactobacillus salivarius* clade. Appl Environ Microbiol 81:1297–1308. doi:10.1128/AEM.03594-14. 25501479 PMC4309690

[6] Klein G, Dicks LM, Pack A, Hack B, Zimmermann K, Dellaglio F, Reuter G 1996 Emended descriptions of *Lactobacillus sake* (Katagiri, Kitahara, and Fukami) and *Lactobacillus curvatus* (Abo-Elnaga and Kandler): numerical classification revealed by protein fingerprinting and identification based on biochemical patterns and DNA-DNA hybridizations. Int J Syst Evol Microbiol 46:367–376.

[7] Nakano K, Shiroma A, Tamotsu H, Ohki S, Shimoji M, Ashimine N, Shinzato M, Minami M, Nakanishi T, Teruya K, Satou K, Suzuki C, Kimoto-Nira H, Kobayashi M, Mizumachi K, Aoki R, Miyata S, Yamamoto K, Ohtake Y, Eguchi-Ogawa T, Moriya N, Hagi T, Nomura M, Hirano T 2016 First complete genome sequence of the skin-improving *Lactobacillus curvatus* strain FBA2, isolated from fermented vegetables, determined by PacBio single-molecule real-time technology. Genome Announc 4(5):e00884-16. doi:10.1128/genomeA.00884-16. 27587811 PMC5009968

[8] Jo SG, Noh EJ, Lee JY, Kim G, Choi JH, Lee ME, Song JH, Chang JY, Park JH 2016 *Lactobacillus curvatus* WiKim38 isolated from kimchi induces IL-10 production in dendritic cells and alleviates DSS-induced colitis in mice. J Microbiol 54:503–509. doi:10.1007/s12275-016-6160-2. 27350616

[9] Lucquin I, Zagorec M, Champomier-Vergès M, Chaillou S 2012 Fingerprint of lactic acid bacteria population in beef carpaccio is influenced by storage process and seasonal changes. Food Microbiol 29:187–196. doi:10.1016/j.fm.2011.08.001. 22202872

[10] Zerbino DR 2010 Using the Velvet *de novo* assembler for short-read sequencing technologies. Curr Protoc Bioinform Chapter 11:Unit 11.5. doi:10.1002/0471250953.bi1105s31. PMC2952100 20836074

[11] Darling AE, Mau B, Perna NT 2010 progressiveMauve: multiple genome alignment with gene gain, loss and rearrangement. PLoS One 5:e11147. doi:10.1371/journal.pone.0011147. 20593022 PMC2892488

[12] Vallenet D, Belda E, Calteau A, Cruveiller S, Engelen S, Lajus A, Le Fèvre F, Longin C, Mornico D, Roche D, Rouy Z, Salvignol G, Scarpelli C, Thil Smith AA, Weiman M, Médigue C 2013 MicroScope—an integrated microbial resource for the curation and comparative analysis of genomic and metabolic data. Nucleic Acids Res 41:D636–D647. doi:10.1093/nar/gks1194. 23193269 PMC3531135

[13] van Heel AJ, de Jong A, Montalbán-López M, Kok J, Kuipers OP 2013 BAGEL3: automated identification of genes encoding bacteriocins and (non-)bactericidal posttranslationally modified peptides. Nucleic Acids Res 41:W448–W453. doi:10.1093/nar/gkt391. 23677608 PMC3692055

[14] Siezen R, Boekhorst J, Muscariello L, Molenaar D, Renckens B, Kleerebezem M 2006 *Lactobacillus plantarum* gene clusters encoding putative cell-surface protein complexes for carbohydrate utilization are conserved in specific gram-positive bacteria. BMC Genomics 7:126. doi:10.1186/1471-2164-7-126. 16723015 PMC1534035

[15] Chaillou S, Champomier-Vergès MC, Cornet M, Crutz-Le Coq AM, Dudez AM, Martin V, Beaufils S, Darbon-Rongère E, Bossy R, Loux V, Zagorec M 2005 The complete genome sequence of the meat-borne lactic acid bacterium *Lactobacillus sakei* 23K. Nat Biotechnol 23:1527–1533. doi:10.1038/nbt1160. 16273110

